# Nanoscale Molecular Characterization of Hair Cuticle Cells Using Integrated Atomic Force Microscopy–Infrared Laser Spectroscopy

**DOI:** 10.1177/0003702820933942

**Published:** 2020-10-06

**Authors:** Alexander P. Fellows, Mike T. L. Casford, Paul B. Davies

**Affiliations:** Department of Chemistry, University of Cambridge, Lensfield Road, Cambridge, UK

**Keywords:** Proteins, lipids, cuticle sub-structure, atomic force microscopy-infrared spectroscopy, AFM-IR, nanometer IR mapping

## Abstract

The hair cuticle provides significant protection from external sources, as well as giving rise to many of its bulk properties, e.g., friction, shine, etc. that are important in many industries. In this work, atomic force microscopy-infrared spectroscopy (AFM-IR) has been used to investigate the nanometer-scale topography and chemical structure of human hair cuticles in two spectral regions. AFM-IR combines atomic force microscopy with a tunable infrared laser and circumvents the diffraction limit that has impaired traditional infrared spectroscopy, facilitating surface-selective spectroscopy at ultra-spatial resolution. This high resolution was exploited to probe the protein secondary structures and lipid content, as well as specific amino acid residues, e.g., cystine, within individual cuticle cells. Characterization across the top of individual cells showed large inhomogeneity in protein and lipid contributions that suggested significant changes to physical properties on approaching the hair edge. Additionally, the exposed layered sub-structure of individual cuticle cells allowed their chemical compositions to be assessed. The variation of protein, lipid, and cystine composition in the observed layers, as well as the measured dimensions of each, correspond closely to that of the epicuticle, A-layer, exocuticle, and endocuticle layers of the cuticle cell sub-structure, confirming previous findings, and demonstrate the potential of AFM-IR for nanoscale chemical characterization within biological substrates.

## Introduction

The primary importance of hair for mammals is in providing thermal insulation. Additional benefits include protecting the skin against harmful ultraviolet (UV) radiation and facilitating cooling through perspiration.^1–3^ Therefore, understanding the physical and chemical structure of hair fibres is crucial for maintaining its desirable properties and hence it has been the focus of much research for over 150 years.^4^

**Figure 1.**
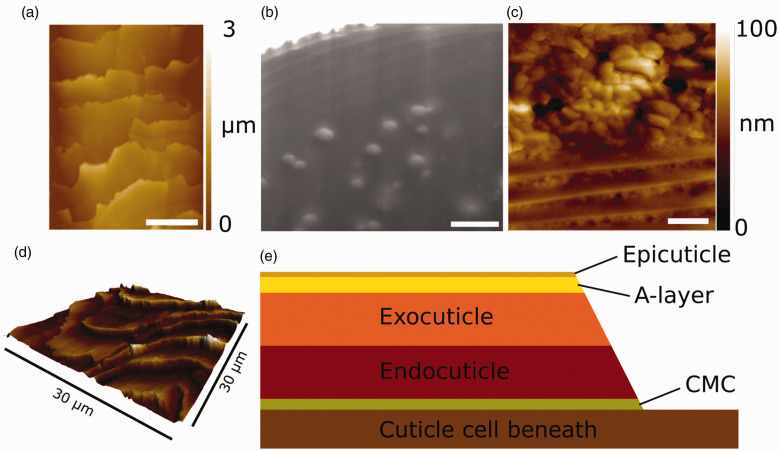
Cuticle structure showing (a) AFM topography across the external hair surface, (b) SEM image of a sectioned hair showing six overlapping cuticle cells and a region of the cortex, (c) AFM topography of a hair cross-section showing the layered structure within each cuticle cell as well as the overlapping cells that make up the cuticle, (d) 3D topography of the surface showing the overlapping and splayed-out nature of the cuticle cells, and (e) diagrammatic representation of the layered sub-structure of the cuticle cell. The scale bars in (a)–(c) represent 10, 1, and 1 µm at the surface, respectively.

The hair fiber cross-section consists of three main regions, the medulla, the cortex, and the cuticle.^5^ The innermost region, the medulla, comprises vacuolated cells distributed along the center of the fiber that are bound together within a matrix and held by the surrounding cell membrane complex (CMC).^6^ The CMC consists of a central proteinaceous and polysaccharide-rich δ-layer, surrounded by an inner and outer lipid rich β-layer, the most prevalent of which is 18-methyleicosanoic acid (18-MEA).^7–9^ The majority of the hair is made up of the cortex which consists of tightly packed, long cortical cells that are aligned parallel to the hair fiber and embedded within corticular CMC.^3,10^ The outermost region of the hair fiber comprises cuticle layers which serve as a protective outer film consisting of 5–10 overlapping scales (cells), each approximately 0.5 to 1.0 µm thick yielding a total thickness of ∼2.5–10 µm for the cuticle. Individual cuticle cells have an internal layered structure consisting of the epicuticle, A-layer, exocuticle and endocuticle, and are separated from each other by cuticular CMC.^3,10–13^ The structure of the cuticle is shown in Fig. 1 where external atomic force microscopy (AFM) mapping across the fiber (Fig. 1a) demonstrates the lateral arrangement of cells, and scanning electron microscope (SEM) imaging of a sectioned hair (Fig. 1b) shows the overlapping cuticle cells that constitute the cuticle. AFM mapping of a cross-section (Fig. 1c) also shows the overlapping cuticle cells but additionally demonstrating the layered sub-structure to individual cuticle cells. Furthermore, a 3D representation of the external surface mapped by AFM (Fig. 1d) demonstrates the “splayed-out” nature of the cuticle cells that results from the growth process. Finally, a schematic of the layered sub-structure of individual cuticle cells is given in Fig. 1e. More detailed imaging through electron microscopy of cuticle cells can be found elsewhere in the literature.^14^

The outermost layer of the cuticle, the epicuticle, has been shown to be approximately 13 nm thick and consist mainly of protein (∼80%), with small amounts of lipid and no evidence of carbohydrate.^11,15,16^ In addition, the external surface of the outermost cuticle is covered by a lipid β-layer (18-MEA), originating from the CMC (where the δ- and second β-layer are no longer present).^7^ The layer beneath, the A-layer, provides significant physical and chemical resistance to external stimuli due to its high cystine concentration (the oxidative product of the combination of two cysteine molecules containing a S–S linkage) and significant proportion of β-sheet protein structures, hence containing extensive cross-linking.^14^ This layer is therefore high in protein content with only small amounts of lipid.^17,18^ The thickest layer, the exocuticle, lies beneath the A-layer and also has a significant protein network and high cystine concentration. However, at only 15% cystine, the lower concentration compared to the A-layer results in less cross-linking and a smaller, but still significant, physical and chemical resistance.^19^ The final, innermost, layer is the endocuticle which is high in protein but with only 3% cystine and does not contribute much to the resistive properties of the cuticle.^19^

The CMC separating individual cuticle cells binds them together. As mentioned above, the lipid 18-MEA is also the major constituent of the external β-layer, where it covalently binds the S-moieties in the epicuticle and provides the first defensive barrier against external agents.^7–9,20^ This lipid layer has been the focus of much research since it is not only a protective barrier but also affects the physical properties of the external surface of hair like smoothness and hydrophobicity.^21,22^ In general, characterizing the physics and chemistry of the cuticle has been of particular importance in research because it provides the fundamental protection and insulation properties of hair.

Previous work using vibrational spectroscopy to study hair structure, such as ATR and Fourier transform infrared (FT-IR) and Raman spectroscopies, provided bulk spectra with some depth profiling of the fibre.^23,24^ Chemical microscopies, e.g., confocal Raman, have been applied to determine chemical variation through the hair fiber using microtome cross-sections.^25^ These methods yield relatively poor spatial resolution however, owing to the diffraction limit of light, and thus cannot reveal many crucial sub-micron features of the fiber. In contrast, AFM-IR is a hybrid technique that integrates established atomic force microscopy (AFM) with a tunable infrared (IR) laser to yield surface selective spectral mapping at nanometer resolution. It works by using the AFM tip to detect the localized thermal expansion that follows IR absorption by a vibrational transition of surface molecules and therefore can circumvent the diffraction limit.^26^ In earlier work, Marcott et al. used AFM-IR to show localization of structural lipids in the medulla and cuticular CMC between individual cuticle layers using maps of the ratio of lipid (using 2924 and 2930 cm^−1^ bands corresponding to aliphatic methylene stretching modes) to keratin proteins (2960 cm^−1^ methyl asymmetric stretch, and 1525 cm^−1^ amide II band).^27^ The current work aims to characterise the chemical structure of the cuticle itself using the high spatial resolution of AFM-IR, focusing on the amide I, lipid carbonyl and cystine-related bands.

## Methods

### Sample Preparation

Human hairs (European brown), obtained from multiple individuals, were washed several times with Millipore water and, following drying, mounted on steel substrates with double-sided tape for AFM-IR analysis of the cuticle cells.

### AFM-IR Analysis

Spectra and maps were recorded in contact mode using an Anasys NanoIR2 instrument equipped with a MIRcat Laser system (Daylight Solutions) containing four quantum cascade lasers (QCLs) covering the 1125–2298 cm^−1^ spectral range. The AFM cantilever-tip assemblies used were gold-coated with 30 nm tip radius, spring constant 0.07–0.4 nm^−1^ and resonant frequency 13 ± 4 kHz (Anasys Instruments). Mapping was achieved with a 0.5 Hz scan rate at resolutions of at least 200 × 200 pixels. IR analysis was done in resonant-enhanced mode (using a 3% duty cycle) with mapping tracking the contact resonance with a phase-locked loop (PLL). Laser powers ranged between 8% for traversing the cuticle cell edge and 30% for probing the external heterogeneity. Spectra were recorded at 1 cm^−1^ per point to enable optimum subtraction from sharp water vapor contributions. Transitions between lasers were largely avoided by analyzing spectral regions covered by a single laser, except for the transition within the carbonyl stretching region which was corrected using step correction.

### Spectral Analysis

**Figure 2.**
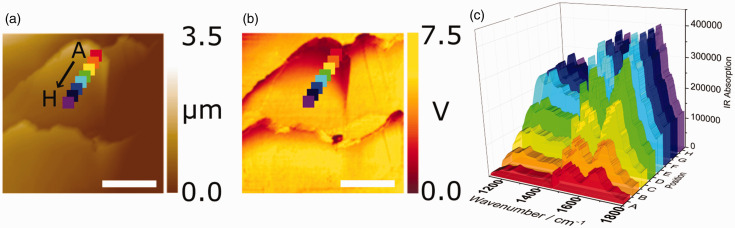
AFM-IR maps and spectra of the external surface of an untreated European brown hair showing (a) height topography, (b) IR intensity at 1630 cm^−1^ corresponding to the amide I band, (c) line profile spectra for positions A–H as indicated by markers in (a) and (b). Position A is closest to the scale-edge moving incrementally away from the edge to position H. The scale bar represents 5 µm across the surface.

AFM-IR spectra were recorded under a reduced-humidity (dry N_2_) environment to minimize water vapor contributions that can otherwise dominate the amide I region. Resulting background-subtracted spectra are shown having been subsequently smoothed either using a second-order Savitzky–Golay algorithm for a cosmetic noise reduction (Figs. 2 and 6) or using a low-pass fast Fourier transform (FFT) filter to remove high-frequency noise contributions while minimizing the effects on the underlying spectral features (this treatment was carefully applied by comparing with raw spectra to minimize spectral deviations, a demonstration of this method is given in Supplemental Material, Fig. S1). The latter smoothing algorithm enables derivative analysis for band-center identification and hence deconvolution into the contributing bands. The spectral baselines were observed to scale with intensity and hence showed no other significant alterations between spectra. Spectra were not baseline-corrected because of the uncertainty associated with selecting a baseline. The marginal baseline variations were estimated and incorporated into the given uncertainties for relative band intensities.

Spectra were recorded across at least five different cuticle cells from at least five different hairs. There were significant differences between samples in terms of relative variations; however, the differences were qualitatively reproducible. The presented spectra and analysis are from a representative sample.

## Results

### Cuticle Surface Approaching the Scale-Edge

Initially, any lateral heterogeneity in the cuticle structure was probed by top-down mapping across the external cuticle cell surface with AFM-IR. Figure 2a shows a representative topographical map of the overlapping scale structure of the cuticles on the hair surface. Figure 2b is the complementary infrared intensity map at 1630 cm^−1^, corresponding to the β-sheet amide I band that is a dominant protein within the cuticle, showing that the intensity decreases towards the edge of the cuticle cell. This drop in intensity arises either because of a lower β-sheet protein secondary structure concentration towards the edge of the cell, or because the IR laser penetrates less deeply into the cuticle leading to a lower sampling depth. Two less likely possibilities that could lead to the decreased intensity were considered, based on technical details associated with recording AFM-IR maps. The first stems from the resonant enhancement that was employed, whereby the QCL laser frequency is tuned to match that of the resonant frequency of the cantilever. This resonant frequency can, however, vary due to any changing mechanical properties of the surface. This can be monitored by continuously tracking the phase and live resonance using a PLL. Inadequate tracking of the contact resonance using the PLL, however, can result in significant intensity artifacts. Furthermore, mechanical differences in the surface may result in a broadening and concurrent intensity decrease to the contact resonance that, even if perfectly tracked by the PLL, will result in a lowering of the observed IR intensity for a given laser power. Both these possibilities can be discounted since the observed changes to the contact resonance frequency were small (as shown in Supplemental Material, Table S1) and the PLL appeared to track these efficiently. Additionally, the apparent loss in intensity can also be seen in the spectra (Fig. 2a) for which the laser is tuned to the contact resonance at each surface location.

To determine the origin of the spectral intensity variation, a series of survey spectra were recorded from the edge of the cuticle cell inwards at the markers indicated in Fig. 2a and b (positions A to H where A is closest to the edge of the cuticle cell). Figure 2c shows the individual spectra where it can be seen that there is an intensity decrease across the whole spectrum for positions closer to the edge, showing that the observed drop in intensity in the AFM-IR map at 1630 cm^−1^ (Fig. 2b) nearer the cuticle cell edge is due to an overall decrease in intensity rather than a specific decrease of the amide I β-sheet band contribution. Such an intensity variation clearly dominates any spectral variations to the IR mapping; hence, further analysis of any lateral inhomogeneity to the chemical composition requires comparison of localized point spectra. It has been shown that changes in protein secondary structures cause significant variation in the amide bands and in particular in the amide I band due to the different environment-dependent contributions of the amide groups in each secondary structure.

**Figure 3.**
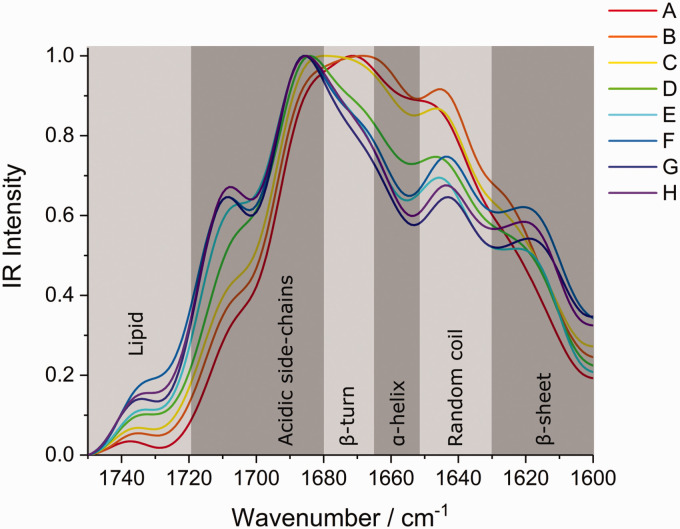
Normalized FFT-filtered AFM-IR spectra in the region of the amide I band at positions A to H. The spectral regions corresponding to the different constituents (as assigned in Table 1) are indicated for identification of the compositional variations.

**Figure 4.**
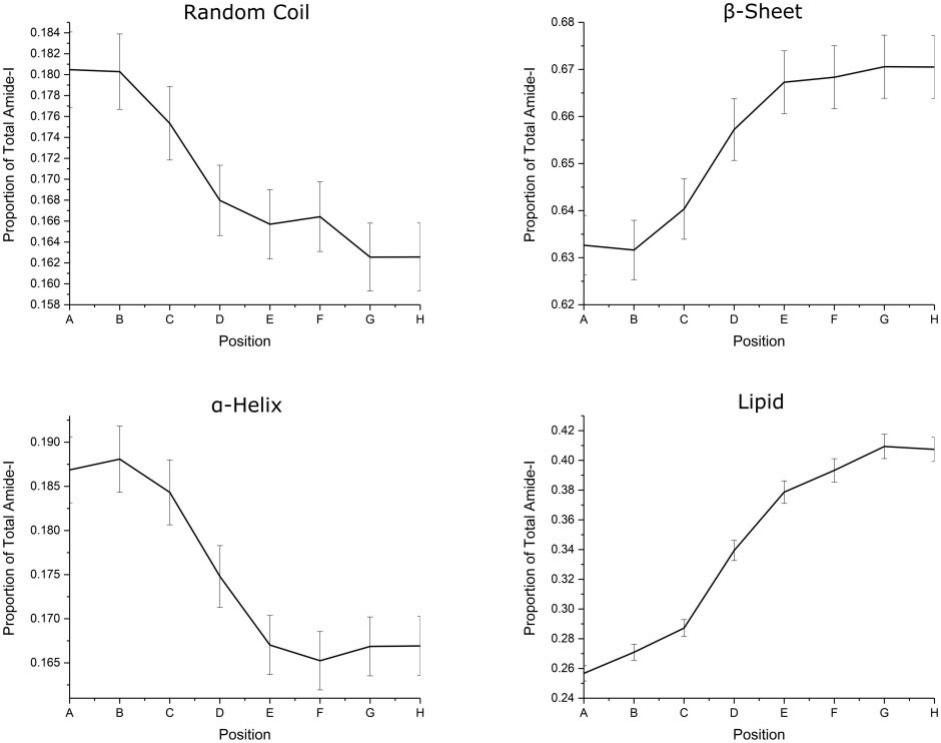
Variation in protein secondary structures and lipid as a proportion of the amount of total amide I (determined from the total band intensity contribution to the amide I and lipid regions) for positions A to H. Uncertainties are estimated using the spectral noise and background variations resulting in the uncertainties associated within relative band contributions.

Subsequently, the recorded spectra from Fig. 2 were used to determine any changes to protein secondary structures and/or lipid contributions on approaching the edge of the cuticle cell. In order to minimize the contributions from high-frequency ambient noise or any residual water vapor (still present due to incomplete purging with dry nitrogen and nonlinearity of contributions in photothermal spectroscopy), spectra were treated with a low-pass FFT filter. Such treatment was observed to minimize noise contributions while maintaining the underlying spectral contributions, as demonstrated in Fig. S1, Supplemental Material (with laser power background showing the water vapor contributions in Fig. S2). The resulting spectra are shown in Fig. 3 in the amide I and lipid carbonyl stretching regions.^28^ The second and fourth derivative spectra at each of the points A to H are shown in Fig. S3. It is clear in the derivative spectra that each spectrum has the same contributing bands, with minimal shifts in the band origin, and hence the significant changes in the spectra (Fig. 3) are mainly due to the change in relative intensities of the contributing bands. The identified band positions and their proportions relative to the total amide I intensity (determined by the sum of contributing band intensities within this spectral region, calculated from the smoothed spectra shown in Fig. 3) are presented in Table S2. The assignment of each of the bands, based on band positions and literature assignments, is given in Table I.^24,29^ Using these assignments, the spectral regions corresponding to the different protein secondary structures, side chains and lipid species are indicated in Fig. 3 where there are clear variations in the constituents, leading to the observed shift in the amide I band structure. The contributions from specific constituents at each of the positions A to H can be deduced and are presented in graphical form in Fig. 4. These plots show higher proportions of α-helices and random coil structures near the edge (position A) and greater proportions of β-sheets and lipids further inwards (position H). These observations are consistent with the observed changes in Fig. 3, as expected.

**Table I.** Approximate band origins and their assignments in the amide I and lipid spectral region for positions A to H.

Band	Approximate band center (cm^−1^)	Assignment
1	1614	β-sheet
2	1626	β-sheet
3	1644	Random coil
4	1662	α-helix
5	1672	β-turn
6	1689	Asn/Gln Amide side-chains
7	1709	Asp/Glu Acidic side-chains
8	1722	Asp/Glu Acidic side-chains
9	1736	Lipid

The structural features of different protein secondary structures are well known. Specifically, the α-helix consists of an unstretched rod-like coil and, other than in tensile deformation, imparts increased rigidity and stiffness to protein structures.^30,31^ β-sheets, on the other hand, consist of stretched chains hydrogen-bonded into extensive sheets that impart greater flexibility and lower stiffness than the α-helices.^30,31^ Greater proportions of α-helices towards the edge could be suggestive of heterogeneity in the physical and mechanical properties of the cuticle cell, with increased stiffness at the edges. This might be expected owing to the geometrical requirement of high rigidity at the edges to maintain the cell shape (analogous to a cytoskeleton maintaining the cell geometry through increased stiffness). The β-sheet structure, on the other hand, is the natural stable protein conformation and also has been shown to result from applied stress, whereby coils are unraveled into extended chains which subsequently cross-link via H-bonds into the extended sheet structures.^30,31^ The spectral analysis shows that β-sheets become more prevalent further from the edge of the cuticle cells. This suggests that the edge regions are under less stress, indicating that the scales are generally under tension. However, it is also possible that the proteins in the cuticle cells are naturally β-sheet structures due to the fundamental stability of this structure rather than arising from conversion of α-helices due to external stress experienced during the fiber growth phase. It is worth noting that, in all the spectra, the β-sheet contribution is the major component (as expected for spectra dominated by the cuticle) which supports both the conclusion that the scales generally are under tension as well as that the proteins exist in their naturally stable conformation. Further work is required to elucidate the origin of these structures.

The survey spectra in Fig. 2c show an overall decrease in intensity near the cuticle cell edge, which is probably due to lower IR penetration because of the air gap below the cuticle cells, which is greatest at the edge. This means that spectra at the edge are weaker because they arise from the outermost part of the cuticle whereas further in there is the possibility of greater penetration of the IR to lower layers, i.e., deeper cuticle cells and even the cortex. Although attenuation of the laser intensity may be sufficient to excite vibrations deeper into the hair, the resulting photothermal expansion will decay with distance, resulting in greater amplitudes closer to the surface thereby emphasizing the surface selective characteristic of the technique. Nevertheless, although it may be a diminished contribution to the spectrum, it is important to consider the penetration depth and resulting spectral contributions from deeper within the hairs, as well as the effective volume probed at each tip position. The underlying cortex, in its coiled fibrillar state, is generally under fairly low stress and consists predominantly of a α-helix structure (example spectrum given in Fig. S4). Hence, if the spectra of the cuticle cell (in regions away from the edge) had a significant contribution from the underlying cortex, they should show a greater presence of α-helix bands which is not what is observed experimentally (Fig. 4). It is, therefore, concluded that the AFM-IR technique is surface-selective to at least the cuticle region (i.e., ∼2–3 µm effective depth, based on the cuticle thickness demonstrated in the hair cross-sections in Fig. 1) and that contributions to the spectra from below the cuticle are insignificant. The large fall off in intensity near the edge of the cuticle cell compared with the intensity further away from the edge is due to the splayed-out nature of the material at the edge. Away from the edge, the sampling depth is greater than the thickness of one cell, while at the edge, because of the splaying of the cell, only a single cell is sampled. This means that, away from the edge, the spectra are average over several cells, while at the edge sampling occurs over only one cell. Hence, spectra near the edge solely arise from edge contributions, whereas away from the edge, the averaged contributions from several cells will be dominated by non-edge species. The observed variations can therefore be attributed to the difference in these two regions.

Due to the presence of an air gap beneath the cuticle cell, an important consideration in the spectral analysis arises from potential mechanical oscillations of the surface, i.e., the photothermal “drumhead” effect. This would result in a coupling of the photothermal expansion oscillations of the cantilever and those of the mechanical oscillations, resulting in a nonlinear IR response. However, owing to the relatively high stiffness of the cuticle cell, as well as its thickness, the resonant frequency would be significantly smaller than the excitation frequency, as well as the amplitude of any photothermal drumhead effect being small, resulting in negligible motion of the substrate during the IR spectral acquisition. Any coupling of the different oscillations to the cantilever response would be expected to result in significant alterations to the contact resonance of the cantilever which, as previously mentioned, were observed to be small. Hence, any drumhead effect is deemed insignificant in this spectral analysis.

### Cuticle Cell Layered Sub-Structure

**Figure 5.**
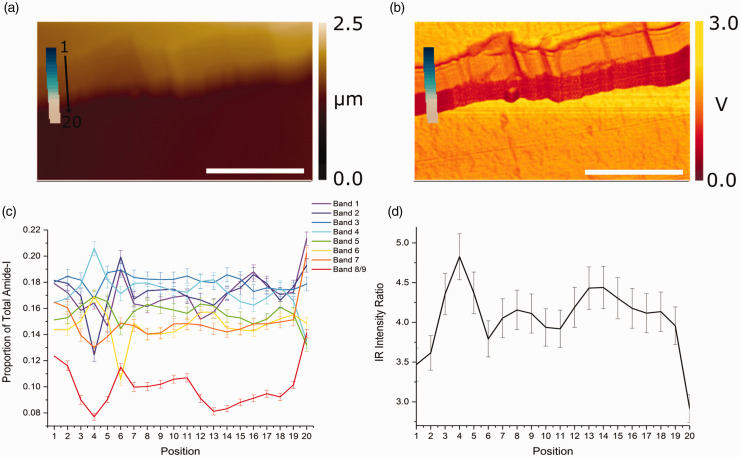
AFM-IR maps and spectral analysis of the cuticle cell edge showing (a) topography, (b) IR intensity at 1650 cm^−1^, (c) spectral profiles from the IR bands identified from the spectra and second derivatives (given in Figure S6), and (d) profile of the IR intensity ratio of bands corresponding to protein to lipid. Markers in (a) and (b) correspond to the positions 1 to 20 (as shown) where spectra were recorded (starting from the top of the image and progressing downwards). The scale bar represents 1 µm at the surface. Uncertainties were estimated allowing for relative spectral noise contributions, background variations and resulting uncertainties in band intensities.

As well as studying the heterogeneity across the cuticle surface, the splayed-out nature of the cells results in their layered sub-structure (as shown in Fig. 1e) being exposed at the edge. AFM-IR was therefore used to examine the chemical variations across the edge that arises from changes between the different layers. Fig. 5 comprises the AFM-IR results for the edge of the cuticle cell, showing maps of topography (Fig. 5a) and IR intensity at 1650 cm^−1^ (Fig. 5b), corresponding to the amide I band from α-helix protein secondary structures. Similar maps showing the layered topography and IR intensity at 1730 cm^−1^, corresponding to the lipid carbonyl stretching bands, can be found in Fig. S5. Although the layered structure is poorly resolved in the topography, it is sharply resolved in the IR intensity maps, indicating that there is significant variation in the chemical structure and, therefore, in the composition of the cuticle cell layers. The observed layers in the chemical map fit well with the known sub-structure to the cuticle, showing two distinct “thick” layers on the inner side, corresponding to the endo- and exocuticle, as well as considerably thinner layers at the outer region, corresponding to the A-layer and epicuticle.

**Figure 6.**
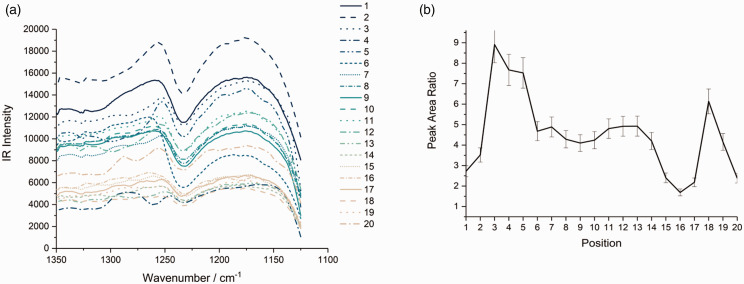
AFM-IR spectra and ratio analysis for the 1125–1350 cm^−1^ spectral region showing (a) spectra corresponding to positions 1–20 as indicated in Figure 5, and (b) cystine-to-protein intensity ratio calculated from the sulfur–oxygen and amide III infrared band integrals.

To further probe the chemical variations across the edge, a spectral line array was recorded at the points indicated by the markers in Figs. 5a and b. The individual spectra, labelled subsequently from 1 to 20, commence at the outermost cell surface of the cuticle and end on the external surface of the cell beneath (i.e., traversing the edge). The recorded spectra and their second derivatives are shown in Fig. S6. The deconvoluted band origins and their corresponding intensities are presented in Table S3. Fig. 5c shows the intensity variations with position traversing the edge for each contributing band. Linking the observed layers to the positions where individual spectra were recorded (positions 1–20) yields a close correlation between changes in the spectral fingerprint and the observed layered structure of the cuticle cell.

It is instructive to examine the ratio of the protein to lipid contributions which can be calculated using the amide I bands and ester carbonyl stretch, respectively, as shown in Fig. 5d. Starting at position 1, this shows a lower protein contribution, and conversely a higher lipid concentration, rising to a maximum at position 4. This subsequently drops away remaining roughly constant until position 11 after which it increases again up to position 14. Finally, it starts to decrease again until suddenly dropping at position 20. These observed qualitative variations are consistent with the known relative protein and lipid concentrations within the different layers that make up the cuticle cell, as previously discussed.

Probing the edges of the cuticle cell presents similar problems regarding penetration depth and relative molecular contributions to those arising from probing the top of the cuticle cell surface. This is because the tip probing the edge is not exactly perpendicular to the edge surface. If it was then the tip would probe only a single layer, e.g., the exocuticle, endocuticle, etc., and the structure deduced from the spectroscopic result would be unambiguous. However, the deviation from normal incidence means that, starting at the top of the cuticle edge, the tip scans not only the first layer but also the layers lying beneath it. As the tip traverses downwards, it continues to record contributions from layers below the layer on which it is positioned, but not from any layers above, e.g., if the tip is moved from being positioned on the exocuticle to the endocuticle, then the exocuticle no longer contributes to the spectrum. The contributions at the endocuticle position, therefore, arise mainly from this layer and from smaller contributions from the CMC beneath, determined by the attenuation of the beam and decay in the associated thermal expansion amplitude in each layer. This emphasizes the surface selectivity of the technique: the layer immediately adjacent to the tip makes the greatest contribution. The clear layered structure of the AFM-IR map in Fig. 5b is a consequence of this, namely, if the surface contribution was not dominant, the IR intensity would be expected to diminish evenly with thickness, which is not what is observed in practice. In summary, the absence of contributions from layers above the tip position and the relative importance of the layer directly next to the tip validates the spectral comparisons made between the different layers present at the edge of a single cuticle cell.

Initially, the AFM tip probes the outer β-layer that is high in lipid content (18-MEA) giving the low protein-to-lipid ratios found for positions 1 and 2. The higher ratio at positions 3 to 5 corresponds to the higher protein content of the epicuticle and underlying A-layer. The exocuticle begins at position 6, where the protein content drops. This lower protein content lasts until position 12/13 which corresponds to the clear boundary between the two thick layers in Fig. 5b. This is apparent in the IR intensity map in Fig. 5b which marks the transition from the exocuticle to the endocuticle. After this boundary, the protein content rises. From positions 14–19, the ratio appears to fall slowly until it abruptly drops at position 20 as the tip probes the β-layer of the next cuticle cell. The gradual drop in protein content after position 14 is likely due to IR penetration through the endocuticle leading to increasing relative contributions from the CMC underneath, which is comparatively high in lipid content.^7–9^

As well as the variable amounts of protein and lipid in the cuticle layers, there is a large difference in the cystine content of the layers which affects the amount of cross-linking of the protein networks. In order to gain insight into the role of cystine and more specifically cystine-related products in the cuticle cell structure, AFM-IR spectra between 1125 and 1350 cm^−1^ were analyzed, corresponding to positions 1 to 20 in Fig. 5, and are presented in Fig. 6a. Although the spectra contain fewer obvious features, second and fourth derivative processing using FFT-smoothed spectra (procedure as used previously) clearly identifies several contributing peaks with the band origins given in Table II. Because the cuticle cells make up the surface of the hair, they are susceptible to oxidation. When this occurs, it converts the cystine into oxidation products like cystine monoxide, cystine dioxide, cysteine-S-thiosulphate and cysteic acid with progressive oxidation eventually leading to cleavage of the cystine S–S bond.^32^ Some of the bands in Table II have been assigned to these oxidation products and provide a measure of the total cystine content.^24,33–37^

**Table II.** Approximate band origins and their assignments in the cystine and amide III spectral region.

Band	Approximate band center (cm^−1^)	Assignment
1	1137	SO_2_ stretch
2	1154	C–N stretch
3	1170	Asymmetric cysteic acid
4	1186	SO_2_ (sulfonyl chloride)
5	1201	Cysteine–S–sulfate
6	1210	S–O stretch/amide III
7	1215	S–O stretch/amide III
8+	>1225	Various amide III secondary structures

The intensity ratio (as used previously for profiles in Figs. 4 and 5) of selected cystine-related bands to selected amide III bands is plotted as a function of positions 1 to 20 in Fig. S7. Bands 1 and 3 in Table II were selected as input for cystine data, chosen as the most prominent bands assigned in earlier work on hair spectra within the 1125–1350 cm^−1^ spectral region, and bands at 1228 and 1242 cm^−1^ for amide III data, because they do not coincide with cystine-related compounds or other features in this spectral region. The most striking feature in Fig. S7 is the significant drop of nearly 10% in the ratio between positions 13 and 18, the region of the endocuticle (clearly identifiable in the IR intensity map in Fig. 5b). This provides unambiguous confirmation of literature reports that the A-layer and exocuticle are relatively cystine-rich, while the endocuticle is cystine-poor. A less prominent cystine-poor region occurs at positions 1 and 2 which agrees with the known composition of the epicuticle and outer β-layer. Interestingly, the apparent increase in cystine content at positions 18 and 19 is actually believed to be due to a contribution from the glycosidic link stretching band in the polysaccharide-rich δ-layer (or similar C–O stretch from triglycerides in this layer) in the underlying CMC that becomes increasingly dominant in its relative contribution at these positions.^38–41^ This increase drops at position 20 since the position 20 spectrum is from the top of another cuticle cell where the dominant contribution arises from the external β-layer and penetration through more of the cuticle layers occurs (as previously discussed).

The significance of the analysis of the data presented in Fig. S7 is reduced by the uncertainties indicated by the error bars on the plot which are larger than those in the analogous plots in Figs. 4 and 5. These uncertainties arise mainly from the noise level in the spectra which therefore results in variations in relative band ratios. The only clear variation in the cystine-to-protein ratio, lying outside the limits imposed by the uncertainties, is the drop in cystine observed between positions 13 and 18 as well as the subsequent increase for positions 18–20 as discussed above. Therefore, to reduce the uncertainties, rather than utilizing specific band contributions, the total band areas were measured by peak integration to give the cystine to protein ratio. Despite the region between 1125 and 1225 cm^−1^ containing some contributions of protein origin, the region above 1225 cm^−1^ contains no cystine-related contributions. Hence, the ratio of the two integrated areas gives an alternative, albeit qualitative, indication of the relative proportions of protein and cystine. This ratio is shown in Fig. 6b where a clear reduction in the magnitude of the uncertainties can be observed. Furthermore, there is a similar ratio profile to that in Fig. S7. This further emphasizes the high cystine concentration in the A-layer (shown here to be even greater than in the exocuticle which could not be distinguished in Fig. S7) with moderate levels of cystine in the exocuticle and subsequent drop for the endocuticle as expected from the known structures of these layers. Again, there is an observed increase in this ratio for position 18 and 19 which is believed to arise from alternative vibrations in the CMC as described above. Finally, the drop at position 20 is observed where the tip is now atop the exposed surface of the cuticle cell beneath and so the ratio here is equivalent to that of position 1 as expected.

## Discussion

The layered cuticle cell structure of the external surface of hair has been widely studied but the current work is the first report of surface-selective infrared spectroscopy at nanoscale resolution. Two spectroscopically important constituents have been investigated yielding fundamental information on the distribution of protein and lipid at the surface. Survey measurements of the cuticle cells using AFM-IR showed significant inhomogeneity to the amide I and lipid carbonyl stretching bands across the top surface of the cell. The spectra show that there is a shift from α-helix and random coil protein secondary structures at the edge to β-sheets further away, although β-sheets remain the prominent structure throughout. β-sheets are known to result from unraveled coils which are under high tension.^30,31^ It is considered that the cuticle cell towards its edges could be under less tension which would explain the lower proportion of β-sheets. In contrast to the proportion of β-sheets, the proportion of α-helices rises towards the edges. α-helices are known to provide greater mechanical rigidity, suggesting that their increasing proportion approaching the edges provides the necessary greater rigidity in these more exposed areas.^30,31^ If there is less tension in the edge region, then random coils would also have a higher proportion there, as found experimentally. An alternative explanation for the differences is that the natural stable protein conformation is the β-sheet structure which dominates from the growth process allowing the more stable structure to form. The change to greater α-helices near the edge, therefore, must result from environmental stresses associated with the edge making the more rigid structure the favored conformation.

The lipid content is also observed to be lower at the edges. The main source of the lipid intensity must be from the external β-layer on top of the epicuticle since the layers below are mainly protein until the CMC is reached at the base of the cuticle cell. As explained earlier, this will only have a weak signal due to the attenuation of the IR intensity by the upper layers as well as the dissipation of the thermal energy deeper inside the hair. The observed loss of lipid towards the edge is expected and consistent with the stripping of the β-layer which commonly occurs in damaged hair.^21,22,42–44^

The partial splaying that occurs at the edge of the cuticle cell, as well as external damage, as mentioned above with regard to the lipid β-layer, provided the opportunity to investigate the layering of the edge of the cuticle cell.^45^ Spectra across the edge, with the tip positioned on individual layers, showed significant changes to the amide, lipid and cystine-related regions. The changes in the spectra revealed a clear layered structure that was consistent with the observed layers in IR intensity mapping, where subsequent interpretation of the spectra confirmed the known proportions of lipid to protein as well as the relative cystine content of the different layers. Specifically, the external surface of the β-layer and epicuticle showed a high lipid and lower protein concentration as well as a lower proportion of cystine. In contrast, the next layer, the A-layer, is higher in protein and lower in lipid along with having a higher cystine component. This agrees with the current view of the molecular composition of these layers.^7,11,15–19^ Lower down below the cuticle cell surface, the AFM-IR spectra provided data on the composition of the exocuticle which showed a high cystine content, although not as large as the A-layer, as well as a slightly increased proportion of lipid. The final layer, the endocuticle, showed a high proportion of protein accompanied by a significant reduction in cystine content. It is proposed that the apparent increase in the cystine content at the bottom of the cuticle is the result of increasing relative contributions from the CMC beneath which provides intensity within this region from other sources. In addition to the spectral investigations of the layers, the physical thicknesses of the observed layers are in accord with other measurements of, for example, the thickest layers, the endocuticle and the exocuticle, as well as the thinner A-layer and epicuticle at the surface.

## Conclusion

The present study has used AFM-IR to investigate spectroscopic aspects of hair cuticle cells, namely the protein and lipid distribution on the cell surface and the protein and cystine composition of the different layers exposed at the cell-edge. As well as investigating changes in the protein and lipid content by analyzing their bands between 1580 and 1750 cm^−1^, cystine containing bands and amide III bands lying between 1125 and 1350 cm^−1^ were also investigated. The composition of the cuticle cell surface showed a shift from higher proportions of α-helices and random coil secondary protein structures at the edge to β-sheets further inwards. Additionally, the lipid composition of the surface also increased on moving away from the edge as expected from the well-known mechanism for physical abrasion of the cuticle. The different chemical composition found for the layered edge of the cuticle cell is compatible with the known cross section of the cuticle cell layers. Initially, the tip probes the outer β-layer which is high in lipid content and low in protein. Below this is the epicuticle and A-layer which are found to have a higher protein content. The protein content drops as the tip probes the next layer, the exocuticle, and finally rises again at the endocuticle, the second of the two thick layers. It was confirmed that the exocuticle layer and A-layer are cystine-rich and the endocuticle layer cystine-poor. These studies demonstrate the potential for AFM-IR to provide significant insight into the nanoscale structures within biological samples and hence emphasize the power of this technique across a broad range of fields.

## Supplemental Material

sj-pdf-1-asp-10.1177_0003702820933942 - Supplemental material for Nanoscale Molecular Characterization of Hair Cuticle Cells Using Integrated Atomic Force Microscopy–Infrared Laser Spectroscopy Click here for additional data file. Supplemental material, sj-pdf-1-asp-10.1177_0003702820933942 for Nanoscale Molecular Characterization of Hair Cuticle Cells Using Integrated Atomic Force Microscopy–Infrared Laser Spectroscopy by Alexander P. Fellows, Mike T. L. Casford and Paul B. Davies in Applied Spectroscopy
