# Supplementary material for: Nanoscale Molecular Characterization of Hair Cuticle Cells Using Integrated Atomic Force Microscopy–Infrared Laser Spectroscopy
Source: Appl Spectrosc. 2020 Oct 6;74(12):1540–50. doi: 10.1177/0003702820933942 (PMC7747034; doi:10.1177/0003702820933942)
Supplement: sj-pdf-1-asp-10.1177_0003702820933942 - Supplemental material for Nanoscale Molecular Characterization of Hair Cuticle Cells Using Integrated Atomic Force Microscopy–Infrared Laser Spectroscopy [file sj-pdf-1-asp-10.1177_0003702820933942.pdf]

Supplemental Material

Nanoscale Molecular Characterisation of Hair Cuticle Cells using Integrated Atomic Force Microscopy–Infrared (AFM-IR) Laser Spectroscopy

Alexander P. Fellows, Mike T. L. Casford,* and Paul B. Davies

Department of Chemistry, University of Cambridge, Lensfield Road, Cambridge CB2 1EW, UK

*Corresponding author email: mtlc2@cam.ac.uk

Table S1. Contact resonance frequencies at points across the cuticle cell surface.

Position	Contact resonance frequency/kHz
A	66.53
B	65.17
C	64.42
D	64.30
E	64.72
F	65.04
G	65.42
H	65.11

Contact resonance peak frequencies for positions A–H as indicated in Figure 2. The uncertainty in the peak frequency is estimated to be ~1-2 kHz, i.e., there is no significant variation with position.

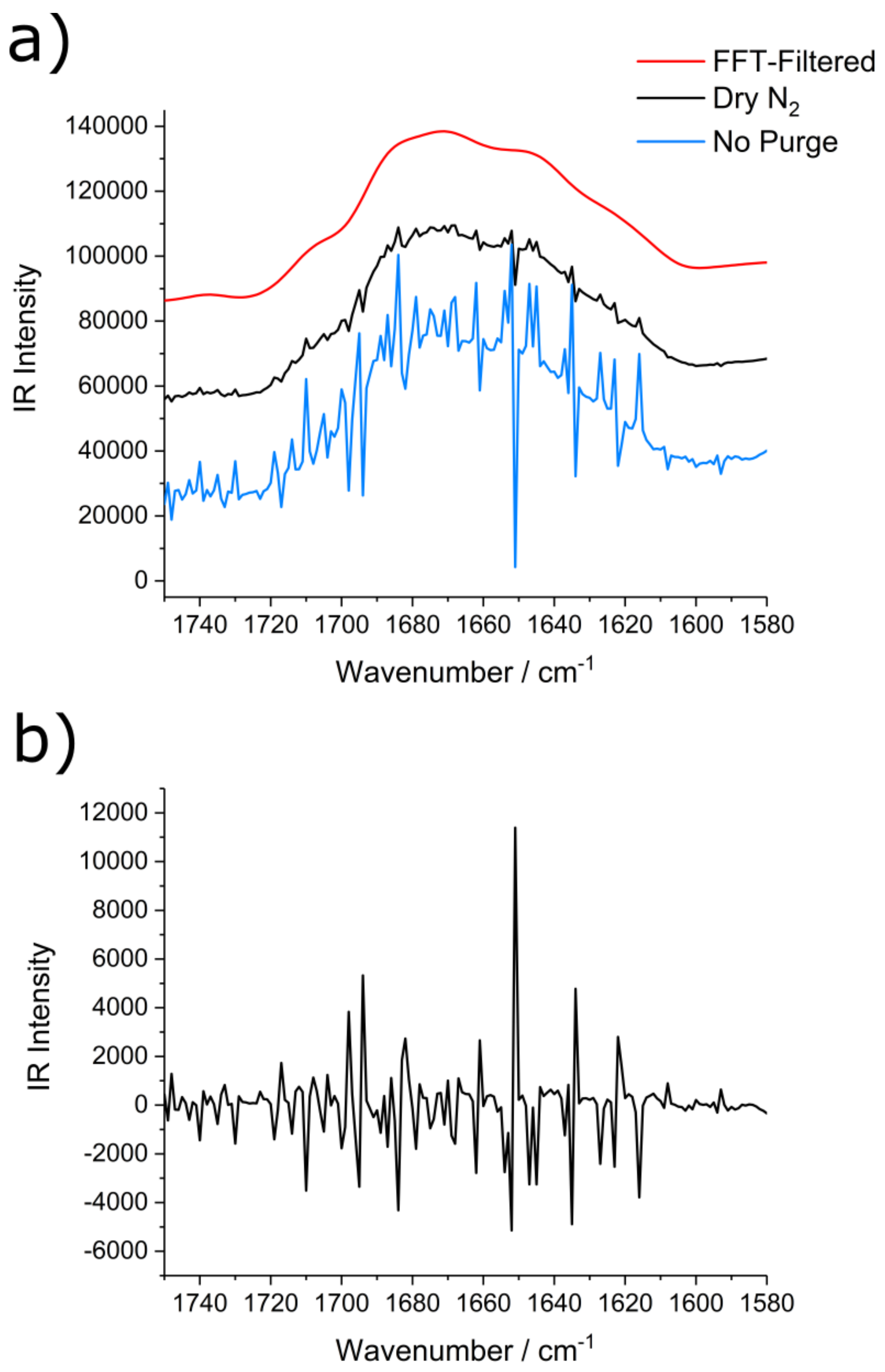

Figure S1. Effect of water vapor on AFM-IR spectra in the amide I and carbonyl stretching region 1580–1750 cm^{-1} showing (a) raw spectra recorded with no purge gas and with a dry nitrogen purge, as well as the FFT-filtered spectrum (b) difference spectrum for the FFT-filtered spectrum and dry purged spectrum in (a).

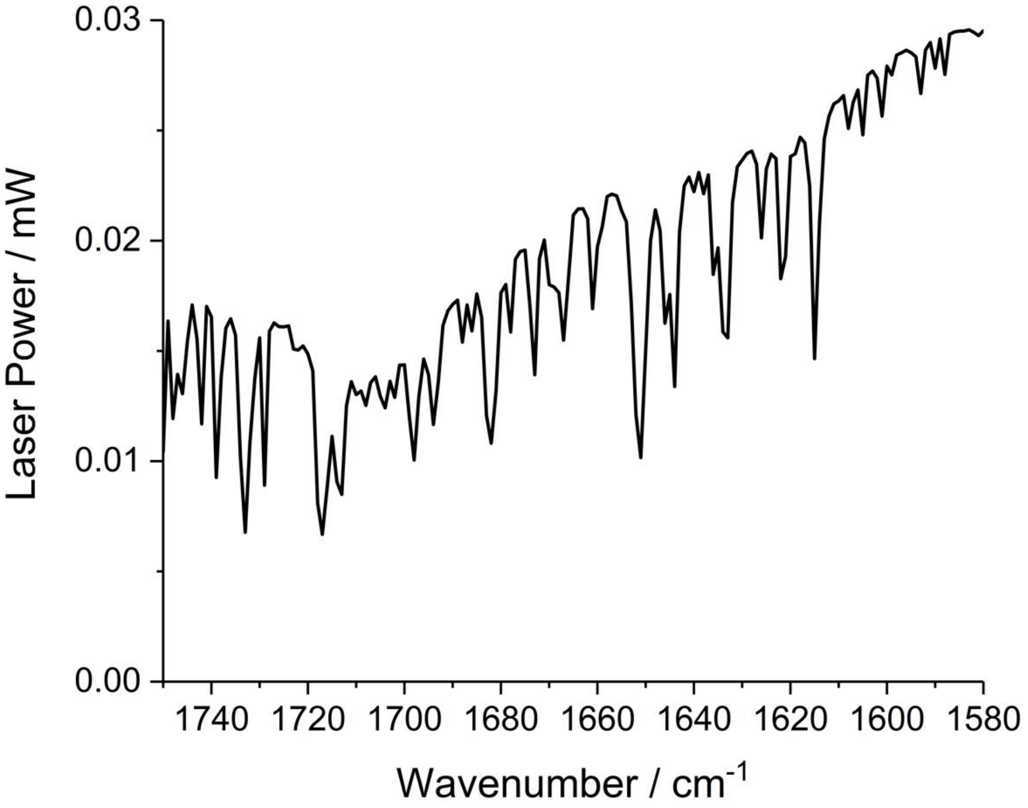

Figure S2. AFM-IR laser background in the 1580–1750 cm^{-1} spectral region without a dry purge. The power spectrum has been step-corrected for the transition between lasers at 1710 cm^{-1} for clarity.

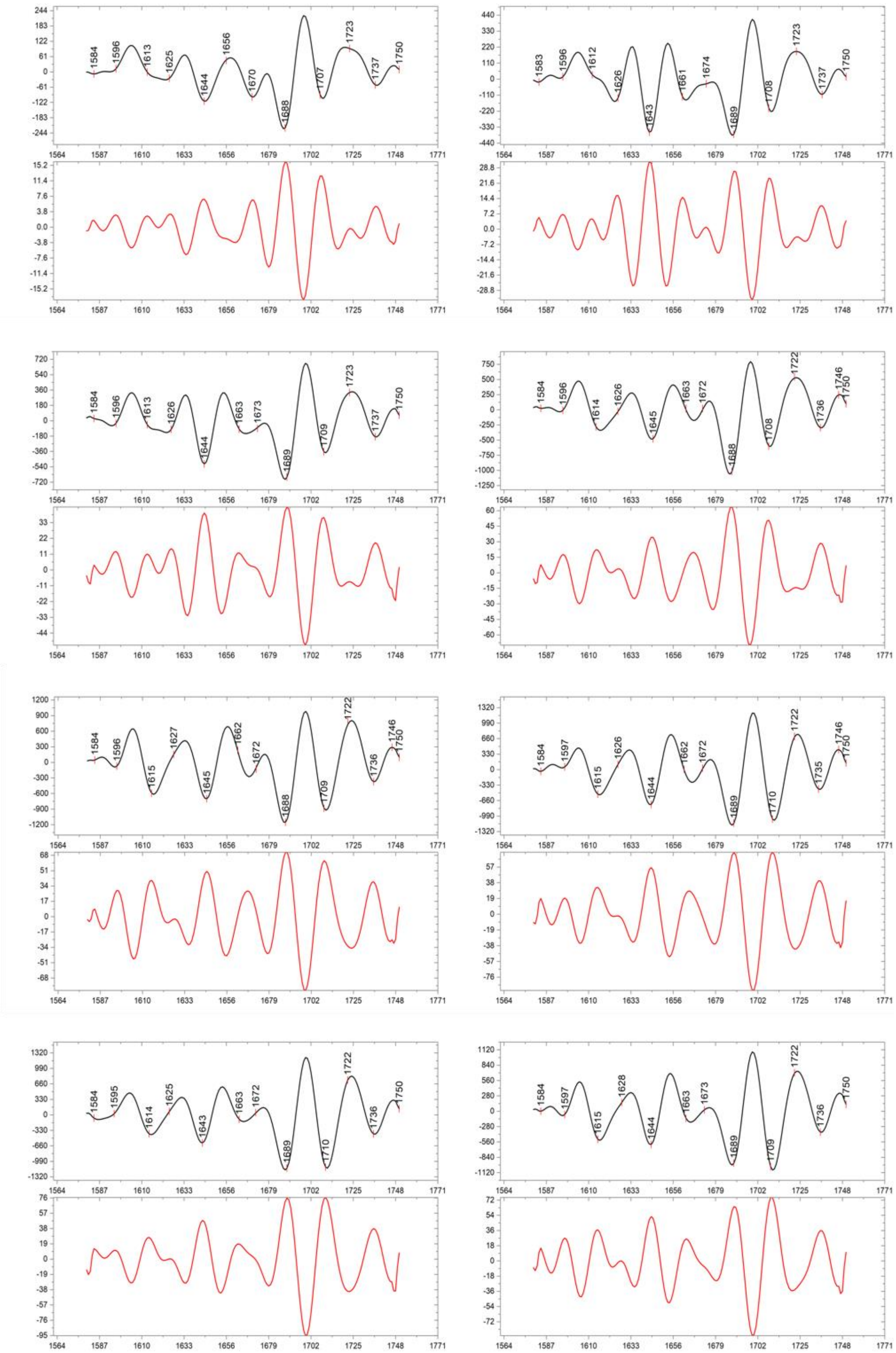

Figure S3. Spectra showing the second (black) and fourth (red) derivatives for positions A-H (ordered in rows) given by the markers in Figures 2a and b (full spectra shown in Figure 2c and spectra for the amide I and lipid region used to calculate the derivatives are shown in Figure 3).

Table S2. Band centers and intensities for the amide I constituent bands for positions A to H.

Position	A	B	C	D	E	F	G	H
Band 1	1613<br>0.113	1612<br>0.118	1613<br>0.123	1614<br>0.133	1615<br>0.142	1615<br>0.151	1614<br>0.151	1615<br>0.152
Band 2	1625<br>0.139	1626<br>0.149	1626<br>0.145	1626<br>0.147	1627<br>0.147	1626<br>0.154	1625<br>0.152	1628<br>0.154
Band 3	1644<br>0.180	1643<br>0.180	1644<br>0.175	1645<br>0.168	1645<br>0.166	1644<br>0.166	1643<br>0.163	1644<br>0.163
Band 4	1656<br>0.187	1661<br>0.188	1663<br>0.184	1663<br>0.175	1662<br>0.167	1662<br>0.165	1663<br>0.167	1663<br>0.167
Band 5	1670<br>0.200	1674<br>0.191	1673<br>0.192	1672<br>0.185	1672<br>0.183	1672<br>0.177	1672<br>0.177	1673<br>0.179
Band 6	1688<br>0.180	1689<br>0.174	1689<br>0.181	1688<br>0.193	1688<br>0.195	1689<br>0.187	1689<br>0.189	1689<br>0.187
Band 7	1707<br>0.112	1708<br>0.116	1709<br>0.121	1708<br>0.143	1709<br>0.157	1710<br>0.156	1710<br>0.162	1709<br>0.162
Band 8	1723<br>0.073	1723<br>0.080	1723<br>0.086	1722<br>0.102	1722<br>0.116	1722<br>0.124	1722<br>0.128	1722<br>0.127
Band 9	1737<br>0.072	1737<br>0.076	1737<br>0.080	1736<br>0.094	1736<br>0.106	1735<br>0.113	1736<br>0.119	1736<br>0.118

The positions (cm^{-1}) and intensities relative to the total amide I intensity of the individual bands in the amide I and lipid spectral region at sampling positions A to H (indicated in Figure 2) on top of and near the edge of the cuticle cell.

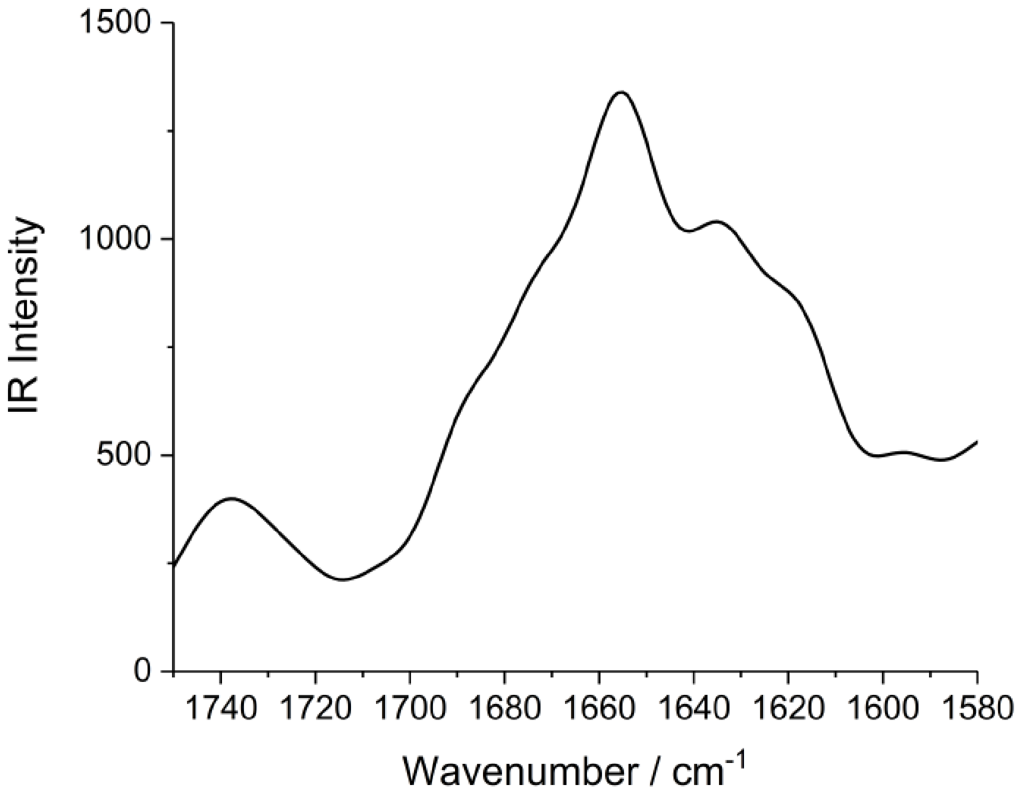

Figure S4. AFM-IR spectrum of hair cortex in the 1580–1750 cm^{-1} spectral range showing the high α-helix contribution at ~1655 cm^{-1}. Spectrum was recorded from a 300 nm cryo-microtomed hair cross section.

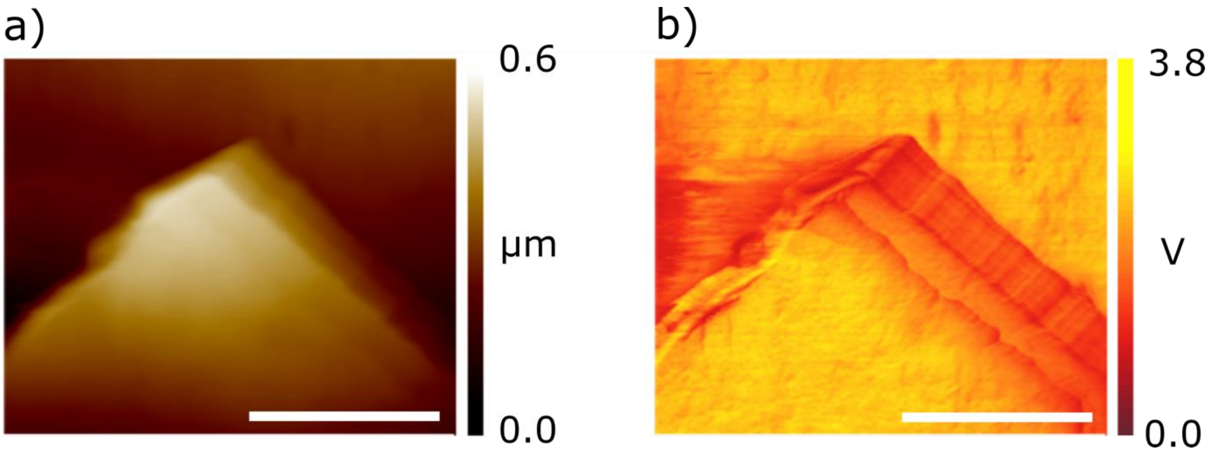

Figure S5. AFM-IR maps at the edge of a cuticle cell showing (a) topography and (b) IR intensity at 1730 cm^{-1}, corresponding to the lipid carbonyl stretching bands. The scale bars represent 1 μm across the surface.

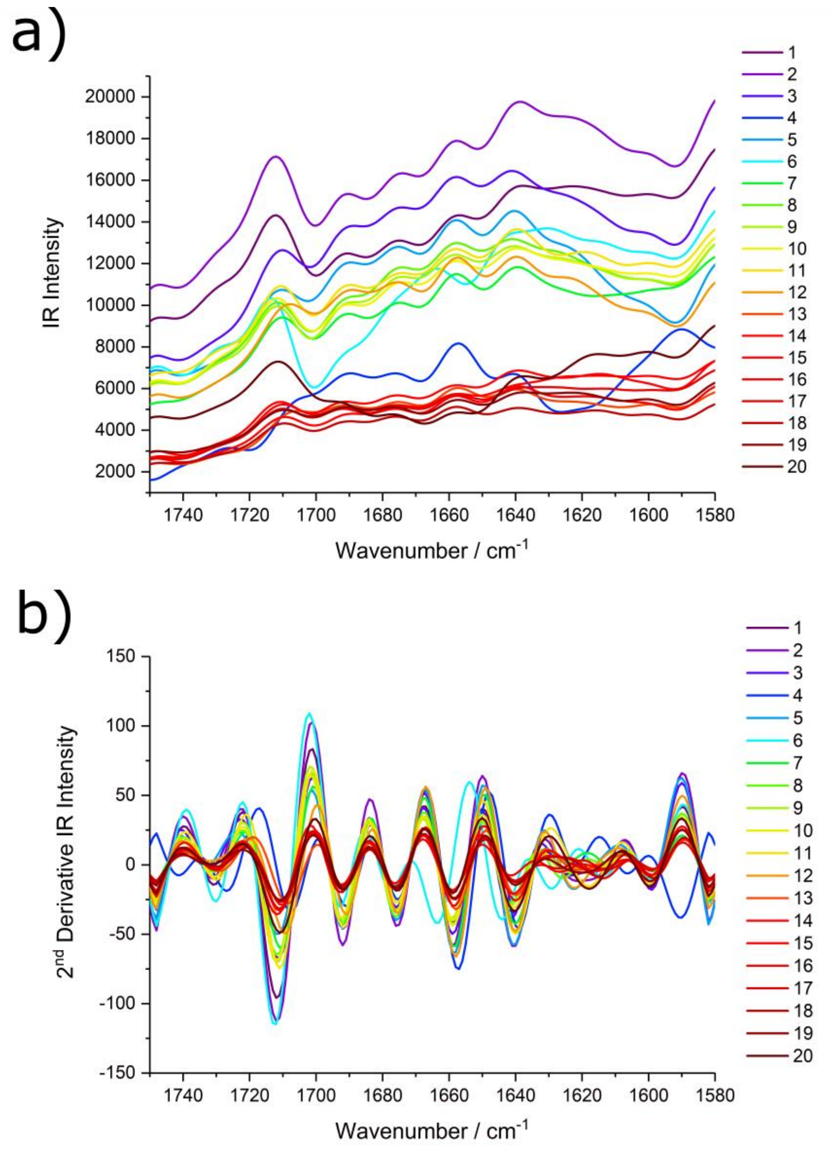

Figure S6. AFM-IR spectra between 1580 and 1750 cm^{-1} at positions 1 to 20 (indicated in Figure 5) showing (a) smoothed spectra and (b) second derivative spectra.

Table S3. The positions (cm^{-1}) and intensity contribution to the total Amide-I intensity of individual bands at positions 1 to 20 across the cuticle cell edge.

Position	Band 1	Band 2	Band 3	Band 4	Band 5	Band 6	Band 7	Band 8/9
1	1614<br>0.179	1625<br>0.181	1641<br>0.180	1659<br>0.165	1676<br>0.151	1692<br>0.144	1711<br>0.165	1731<br>0.124
2	1614<br>0.172	1625<br>0.179	1641<br>0.184	1659<br>0.168	1676<br>0.153	1692<br>0.144	1711<br>0.161	1731<br>0.116
3	1614<br>0.158	1626<br>0.169	1642<br>0.182	1659<br>0.178	1676<br>0.162	1692<br>0.151	1711<br>0.139	1732<br>0.090
4	1607<br>0.164	1623<br>0.124	1639<br>0.167	1657<br>0.206	1675<br>0.169	1691<br>0.169	1708<br>0.130	1729<br>0.077
5	1614<br>0.147	1624<br>0.165	1641<br>0.187	1658<br>0.182	1675<br>0.165	1692<br>0.154	1711<br>0.138	1734<br>0.090
6	1614<br>0.190	1629<br>0.199	1645<br>0.189	1663<br>0.171	1678<br>0.145	1694<br>0.106	1712<br>0.149	1730<br>0.115
7	1612<br>0.163	1625<br>0.167	1641<br>0.184	1658<br>0.179	1675<br>0.158	1692<br>0.149	1710<br>0.147	1729<br>0.100
8	1613<br>0.161	1626<br>0.173	1642<br>0.182	1659<br>0.179	1676<br>0.163	1693<br>0.140	1711<br>0.140	1730<br>0.100
9	1614<br>0.166	1626<br>0.174	1642<br>0.182	1659<br>0.176	1676<br>0.161	1693<br>0.141	1711<br>0.141	1730<br>0.102
10	1613<br>0.169	1626<br>0.175	1641<br>0.182	1659<br>0.174	1676<br>0.159	1693<br>0.142	1711<br>0.148	1730<br>0.106

11	1617<br>0.170	1625<br>0.169	1641<br>0.185	1659<br>0.172	1676<br>0.156	1692<br>0.148	1710<br>0.148	1729<br>0.107
12	1613<br>0.152	1624<br>0.167	1641<br>0.181	1658<br>0.181	1675<br>0.163	1691<br>0.157	1709<br>0.147	1731<br>0.091
13	1614<br>0.157	1623<br>0.163	1641<br>0.180	1658<br>0.182	1675<br>0.162	1691<br>0.157	1709<br>0.144	1732<br>0.081
14	1614<br>0.171	1624<br>0.172	1641<br>0.186	1659<br>0.172	1676<br>0.154	1692<br>0.145	1710<br>0.142	1733<br>0.083
15	1614<br>0.180	1627<br>0.176	1642<br>0.182	1659<br>0.165	1676<br>0.153	1693<br>0.144	1711<br>0.144	1731<br>0.088
16	1613<br>0.188	1627<br>0.186	1642<br>0.173	1659<br>0.162	1676<br>0.148	1693<br>0.143	1711<br>0.148	1730<br>0.091
17	1613<br>0.177	1627<br>0.179	1641<br>0.176	1659<br>0.168	1676<br>0.152	1693<br>0.148	1711<br>0.148	1730<br>0.095
18	1614<br>0.171	1627<br>0.166	1641<br>0.174	1659<br>0.176	1676<br>0.161	1692<br>0.152	1710<br>0.150	1733<br>0.092
19	1616<br>0.172	1624<br>0.177	1641<br>0.174	1658<br>0.166	1676<br>0.156	1692<br>0.155	1710<br>0.151	1729<br>0.101
20	1616<br>0.213	1625<br>0.193	1641<br>0.179	1659<br>0.135	1675<br>0.132	1692<br>0.149	1710<br>0.202	1731<br>0.141

The positions (cm^{-1}) and fractional intensity contribution to the total amide I intensity of the individual bands in the amide I and lipid spectral region at sampling positions 1 to 20 at the edge of the cuticle cell.

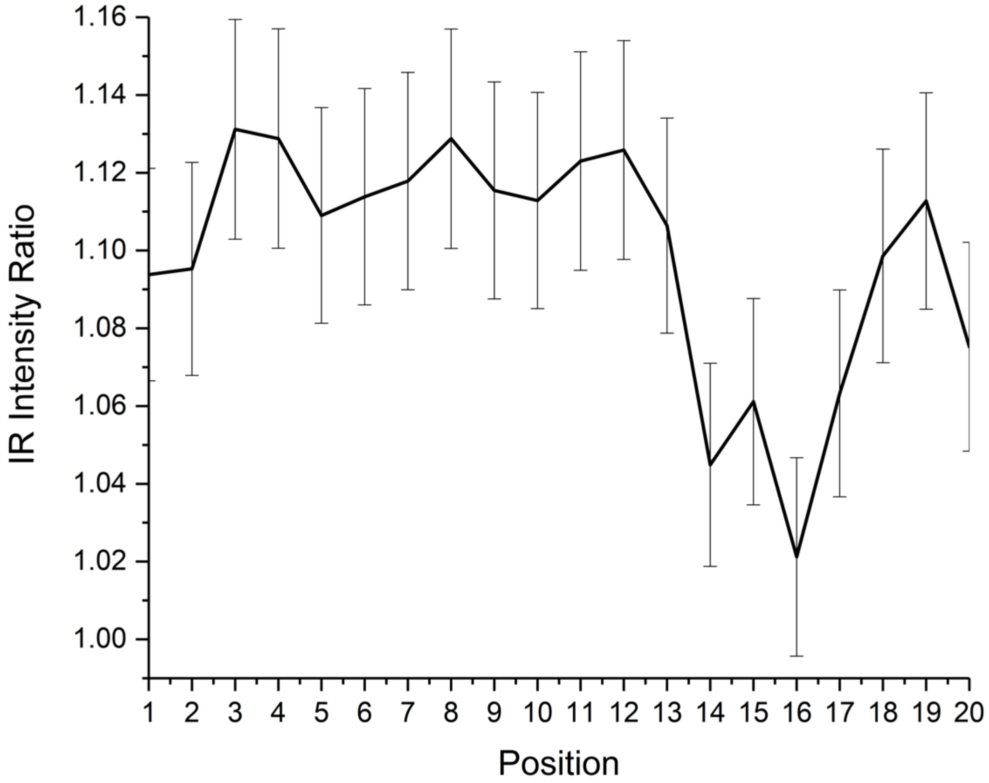

Figure S7. The cystine-to-protein intensity ratio calculated from selected sulfur–oxygen and amide III band intensities as a function of sampling position 1 to 20 (indicated in Figure 5).
